# Experimental Effects of Acute Exercise and Meditation on Parameters of Cognitive Function

**DOI:** 10.3390/jcm7060125

**Published:** 2018-05-29

**Authors:** Meghan K. Edwards, Paul D. Loprinzi

**Affiliations:** Physical Activity Epidemiology Laboratory, Exercise Psychology Laboratory, Department of Health, Exercise Science and Recreation Management, School of Applied Sciences, The University of Mississippi, 229 Turner Center, University, MS 38677, USA; medward3@go.olemiss.edu

**Keywords:** cognition, exercise psychology, meditation, physical activity, walking

## Abstract

Single bouts of aerobic exercise and meditation have been shown to improve cognitive function. Yet to be examined in the literature, we sought to examine the effects of a combination of acute bouts of aerobic exercise and meditation on cognitive function among young adults. Participants (*n* = 66, mean (SD) age = 21 (2)) were randomly assigned to walk then meditate, meditate then walk, or to sit (inactive control). All walking and meditation bouts were 10 min in duration. Participants’ cognition was monitored before and after the intervention using Identification, Set Shifting, Stroop, and Trail Making tasks. Additionally, a subjective assessment of cognitive function was implemented before and after the intervention. Significant group by time interaction effects were observed when examining the Stroop congruent trials (*P* = 0.05). Post hoc paired *t*-tests revealed that reaction time significantly decreased from baseline to post-intervention in both combination groups (*P* < 0.001 for both), but not in the control group (*P* = 0.09). Regarding all other cognitive assessments, there were no significant group by time interaction effects (*P* > 0.05). Cognitive function was not substantially affected by a combination of brief meditation and exercise, though there is evidence to suggest that this combination may have beneficial effects on certain aspects of cognition. Future work should be conducted to evaluate the influences of different doses of exercise and meditation on cognitive functioning.

## 1. Introduction

The numerous academic demands placed on college students warrant a thorough investigation of methods for promoting optimal levels of cognitive functioning. Exercise and meditation are two behaviors that have been examined within such a context. For example, aerobic exercise training has been shown to have favorable effects on cognitive function [1], including acute bouts of aerobic exercise [2,3]. Reviews detailing potential neurobiological mechanisms to explain the relationship between aerobic exercise and cognitive function can be found elsewhere [4,5,6]. Briefly, substantial attention has focused on the effects of acute exercise on brain-derived neurotrophic factor (BDNF) [5], which may help to improve cognitive task performance via (for example) neuronal and synaptic growth as well as long-term potentiation [7]. Previous evidence [8] also supports the ability of mindfulness meditation to improve aspects of cognitive functioning, such as attention- and memory-related parameters. Similar to aerobic exercise, even brief (e.g., four-session) mindfulness meditation training has been found to be effective in improving cognition [9]. Notably, meditation has been shown to activate areas of the brain that are implicated in commonly employed tests of cognitive function (e.g., the anterior cingulate cortex and the prefrontal cortex) [10]. Particularly relevant to the present study, combinations of exercise and mindfulness have been shown to offer cognitive benefits. Mental and physical (MAP) training [11]—a combination of focused attention meditation, a mindfulness-based walk, and a session of submaximal cycling (all performed in succession)—has been shown to reduce ruminative tendencies and improve cognitive control processes among those suffering from depression [12].

The current study was interested in exploring the utility of techniques most feasible to implement among college students. This population is important to study, given that some aspects of cognitive function may start to decline during the early adult years (i.e., 20s) [13]. Roughly based on MAP, the present study used a combination of brief aerobic exercise and a brief open-monitoring mindfulness meditation. Specifically, written herein as a brief report, we were interested in examining whether the order of meditation and aerobic exercise plays a differential role in any observed cognitive outcomes; this potential temporality effect has yet to be examined in the literature. A review detailing focused attention and open-monitoring mindfulness can be found elsewhere [14]. We hypothesized that both combination interventions would result in improvements in cognitive function, but ending a session with exercise (versus ending a session with meditation) would result in greater enhancement of cognition, perhaps through arousal-dependent mechanisms [3]. For example, McMorris’s neuroendocrinological model for exercise-related cognitive benefits [15] suggests that exercise facilitates hypothalamus-induced catecholamine synthesis whereby adrenaline and noradrenaline are released from the adrenal medulla and, subsequently, catecholamines are released in the brain. Norepinephrine and dopamine, for example, are believed to play critical roles in information-processing brain networks. Thus, the main aim of this study was to examine the potential temporal effects of acute meditation and acute walking on cognitive function.

## 2. Methods

### 2.1. Study Design and Participants

A randomized controlled experimental design was employed. After completing baseline surveys and cognitive function assessments, participants were randomized to a “walk then meditate”, a “meditate then walk”, or a control group. Following the intervention, cognitive function was reassessed. Participants were excluded if they (1) were not within the target age range (18–53 years) or (2) had exercised within five hours or consumed caffeine within three hours. 

### 2.2. Walk and Meditation Protocol

Participants were randomized into a walk then meditate (*n* = 22), meditate then walk (*n* = 22), or control (*n* = 22) group. Our employed sample size per group (i.e., 22) is similar to in other related work [16]. The walk entailed self-selecting a brisk walking pace to walk (on a treadmill) for 10 min. The meditation was a 10 min guided, open-monitoring mindfulness meditation. The guided meditation cues focused on breath/body present-moment awareness, limiting mind-wandering/letting go of distractions or worries, and cultivating relaxation. All participants had their eyes closed during the meditation. Participants randomized into the control group were asked to relax and sit quietly (with eyes open) in a computer chair within the laboratory for 10 min. During all protocols, heart rate was assessed via a Polar heart rate monitor (F1, Polar OY, Kempele, Finland). 

### 2.3. Assessment of Cognition

To assess parameters of cognitive function, we utilized both computerized and pen-and-paper tests (in a counterbalanced order). Notably, for all (pre- and post-) tests except for the pen-and-paper listing tasks (detailed below), participants had a practice session prior to completing the scored test. Computer-based tests included CogState’s Identification Task and Set Shifting Task, as well as the Inquisit Stroop Task (Milisecond Test Library). The Identification Task and Set Shifting Task are part of the CogState Brief Battery, which has been validated among samples with varying cognitive abilities [17,18]. The Identification Task measures attention via a choice reaction time paradigm; participants are shown a series of cards and are instructed to select “yes” if the card is red and “no” if it is not. Set Shifting is a test of executive function wherein participants are prompted to indicate whether or not the displayed card is a “target card”. If the screen contains the word “color”, participants are to select whether the displayed card matches the target card color. If the screen contains the word “number”, participants are to select whether the displayed card matches the target card number. After a series of cards, the rule of the test changes (e.g., from one color to the other color). The participant is not informed when the changes occur; thus, part of the test lies in their ability to learn the new rule and proceed quickly. The Inquisit Stroop Task presents 84 total trials, consisting of 4 colors (red, green, blue, black) by 3 color-stim congruencies (congruent, incongruent, control) by 7 repetitions. The stimuli remained on the screen until the key response, with latencies measured from the onset of the stimuli. The congruent trials involved the color word and the color it presented being the same; incongruent trials involved the color word being different than the color it was presented in (e.g., it read GREEN, but this word was not in the green color); and the control trials involved colored rectangles. The outcome measure was the average latency (in milliseconds (ms)) of the correctly identified congruent, incongruent, and control trials. Lower scores indicate better cognitive function. 

Trail Making A and B (pen and paper) were implemented to assess visuoperceptual abilities, working memory, and task switching [19]. For Trail Making A, participants are instructed to connect a series of 25 dots in ascending numerical order. For Trail Making B, participants are instructed to connect a series of 24 dots in alternating ascending numerical order and alphabetical order (e.g., 1, A, 2, B, 3, C). Finally, two timed listing tasks were implemented to assess participants’ perceptions of their cognitive abilities. Subjective assessments of cognition have been implemented in populations that may suffer from cognitive impairments (e.g., individuals with major depression and attention deficit/hyperactivity disorder), with some evidence to suggest that subjective ratings of cognitive abilities are a better indicator of (social, occupational, and psychological) functioning than objective tests of cognition [20]. It seems that objective and subjective assessments of cognition may measure different constructs and thus should both be employed when assessing cognition-related outcomes. Participants were given a minute to write as many numbers as they could, starting at either 2 or 8, and counting up by 7’s (first listing task) and were also asked to list as many female names starting with the first half of the alphabet or as many male names starting with the second half of the alphabet (second listing task) as they could. They were asked to then rate the extent to which they were able to sustain attention, the ease with which they were able to switch between tasks, the creativity they felt they had with listing the names, and their ability to inhibit unsuitable responses during the name listing task (e.g., avoid writing boys’ names or names that start with the first half of the alphabet). Their subjective cognitive function performance scores were provided on a 1–100 rating scale. 

### 2.4. Additional Assessments

Several surveys (see Table 1) were implemented to assess baseline demographic, behavioral, and psychological characteristics across the three groups. Height was assessed using a standard stadiometer and weight was assessed using a standard scale; height and weight were used to calculate body mass index.

### 2.5. Statistical Analysis

Analysis was computed using SPSS software (version 24.0, IBM SPSS, Armonk, NY, USA) and Stata software (version 12.1, Stata Corp LLC, College Station, TX, USA). Demographic differences between the three groups were compared via ANOVA tests of between-group effects for continuous variables and via chi-square tests for any nominal data. To evaluate any potential differential intervention effects, a 3 (groups) by 2 (time) repeated measures ANOVA was employed, with condition serving as the between-subject variable and time (pre/post) as the within-subject variable. Statistical significance was established as *P* < 0.05.

## 3. Results

Descriptive characteristics of the study sample are displayed in Table 1. Demographic comparisons between the three groups revealed that there were no statistically significant differences among the groups with regards to age (mean age = 20.9 years [SD = 2.2]), gender (28.8% male), race/ethnicity (71.2% Non-Hispanic white), BMI (mean BMI = 24.0 kg/m^2^ [SD = 4.3]), physical activity level (mean moderate-to-vigorous physical activity = 294.8 min/week [SD = 255.7]), trait mindfulness, typical responses to stressful situations, emotion regulation, executive function abilities, time since previous meal, exercise enjoyment, or meditation experience.

### 3.1. Manipulation Check

After completing the meditation, participants were provided (via survey assessment) a definition of mindfulness (“Mindfulness is often referred to as a mental state characterized by full attention to internal and external experiences as they occur in the present moment. Mindfulness is additionally characterized by non-judgment of, and openness to current experiences.” [25]). Based on this definition, participants were asked to rate the extent to which they felt they (1) had practiced mindfulness; (2) felt connected to their meditation experience; (3) experienced moments of ‘inner peace’; and (4) were able to return to the meditation experience if they noticed mind-wandering. Response options ranged from 1 (“not at all”) to 4 (“completely”). The average response (1–4) for all four questions was 3.3 (SD = 0.6). For each of the four items, no participants responded with a score of 1. The majority of responses for each of the four questions were 3s or 4s (93%, 88%, 91%, and 86%, respectively). As such, we feel confident that our meditation elicited the intended effects.

Additionally, at the end of the visit, participants were asked to rate the extent to which they enjoyed their meditation or exercise bout. The statements read, “How much did you enjoy your exercise bout?” and “How much did you enjoy your meditation bout?” Response options ranged from 1 “not at all” to 5 “very much”. Nearly all participants rated exercise as 3 or higher, with 84% of participants rating 4 or 5; one participant responded with 1, indicating that he/she did not enjoy their exercise experience. For meditation, 89% of participants rated 4 or 5; 2 participants responded with 2, indicating that these individuals did not enjoy their meditation experiences.

### 3.2. Main Outcomes

**Figure 1:**
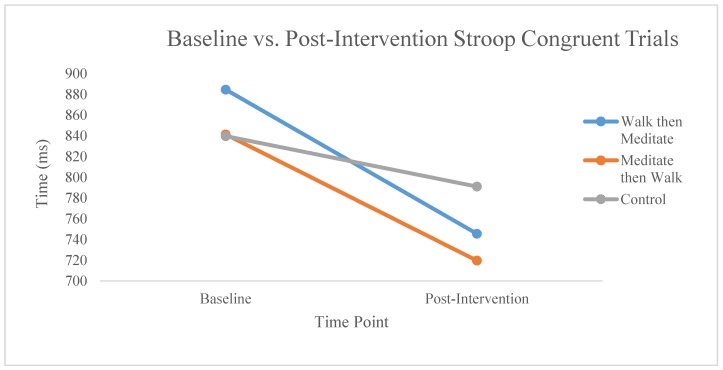
Baseline versus post-intervention Stroop congruent trials.

Table 2 displays the baseline and post-intervention cognition scores. As displayed graphically in Figure 1, a significant group by time interaction effect emerged only for Stroop Task congruent trials (*P* = 0.05). Bonferroni post hoc paired *t*-tests revealed that Stroop scores changed (decreased) significantly from baseline to post-intervention in the walk then meditation (*P* < 0.001) and meditation then walk (*P* < 0.001) groups, but not in the control group (*P* = 0.09). Stroop Task score reductions did not differ significantly between the two intervention groups. Though not shown in Table 2, change scores were calculated for these groups; when comparing walk then meditation versus meditation then walk, there were no significant differences (*P* = 0.66). Additionally, Stroop “interference scores” were calculated. Interference scores resulted in no significant group by time interaction effects (*P* = 0.79). Further, between the three groups, there were no baseline differences in the Stroop Task congruent trials (*F* = 0.30, *P* = 0.74), nor were there any group differences at the post-assessment (*F* = 1.16, *P* = 0.31).

## 4. Discussion

The purpose of this study was to investigate the effectiveness of a combination of 10 min bouts of brisk walking and mindfulness meditation on cognitive function parameters among a sample of college students. Though several tests of cognition were implemented, congruent trials of the Stroop Task yielded the only significant group by time interaction effect. Regarding this interaction, there was no evidence to suggest a significant influence of temporal sequencing for the intervention components; however, both of these temporal combinations resulted in superior Stroop change congruent scores when compared with the control group. Notably, however, differences in the change scores were minimal, as emphasized by the nonsignificant differences in the post-intervention scores among the groups.

Previous work suggests that meditation may benefit various cognitive parameters, even among the aging population as well as those with neurodegenerative diseases [26]. Such cognitive improvements in cognition from meditation have been observed in areas related to attention, memory, verbal fluency, and cognitive flexibility [26]. Mechanisms of this effect are complex and are likely multifaceted, influencing various brain regions via diverse mechanistic pathways [27]. In addition to meditation, acute exercise has also been shown to enhance various cognitive parameters, such as attention, executive function, and memory [28,29,30]. Such effects may occur through various pathways, such as alterations in neuronal excitability as well as proliferation of neurotrophic growth factors [31,32].

Our findings, however, did not provide conclusive evidence of a beneficial effect of meditation and acute exercise on cognition. Our results suggest that combining 10 min of a brisk walk and a 10 min meditation may not offer robust improvements in cognitive function, given that the majority of the employed cognitive tasks did not differ across the intervention arms. Though previous findings have shown positive psychological/cognitive effects to result from a single, brief session of exercise or meditation, these results are in alignment with previous findings. For example, Johnson et al. [33] did not observe significant changes to attention or working memory following a single session of mindfulness meditation among a similarly aged sample. A review published by Chiesa et al. [8] has concluded that the cognitive effects of mindfulness training are most likely related to the amount of meditation training, suggesting that a single session of mindfulness meditation may not be adequate for inducing cognitive changes (especially among nonmeditators). Similarly, previous studies have demonstrated intensity and duration effects related to exercise-induced changes in BDNF [34]. Though our previous findings have demonstrated the effectiveness of a 5 min brisk walk in improving cognitive function [35], it is possible that a 10 min duration was not a sufficient stimulus for robust improvements in cognition. Additionally, our sample was highly active (nearly 300 min/week moderate-to-vigorous physical activity (MVPA)). It is possible that a brisk walk was not a novel enough stimulus to induce noticeable improvements in cognition (previous evidence from our group [36] suggests that students at the researchers’ university walk approximately 9000 steps/day). 

## 5. Conclusions

In conclusion, a 10 min brisk walk in combination with a 10 min meditation did not appear to significantly impact overall cognitive function among an active, young adult population. Beginning a multiweek meditation/exercise intervention may not be a realistic expectation for college students who commonly cite a lack of time for not participating in exercise [37]. Thus, future research should continue to investigate the potential utility of single sessions of combined aerobic training and meditation for enhancing cognition and other psychological parameters. Additionally, further investigation protocols that may positively influence an individual’s subjective assessment of their cognitive abilities may be of value [20]. Given the mixed findings of our current experiment, as well as those of others, future replicative work on this novel paradigm is warranted.

## Figures and Tables

**Table 1:** Demographic characteristics of the analyzed sample (*n* = 66).

	Group			
Variable	Walking + Meditation (*n* = 22)	Meditation + Walking (*n* = 22)	Control (*n* = 22)	*P*-Value ^Ψ^
Age, mean years	21.2 (2.3)	20.7 (1.7)	20.9 (2.5)	0.11
Gender, % male	27.3	22.7	36.4	0.85
Race, %				0.57
Mexican American	0	0	4.5	
Other Hispanic	0	4.5	0	
Non-Hispanic White	77.3	59.1	77.3	
Non-Hispanic Black	13.6	13.6	9.1	
Other	9.1	22.7	9.1	
BMI, mean kg/m^2^	23.1 (4.1)	24.4 (4.2)	24.6 (4.5)	0.64
MVPA, mean min	268.6 (213.4)	287.4 (137.5)	328.5 (416.3)	0.79
Previous meditation experience, %	63.6	50.0	59.1	0.38
Currently meditating, %	36.4	62.5	50.0	0.86
Last meal, mean h	3.8 (3.1)	4.4 (4.7)	4.0 (3.7)	0.82
Dysexecutive function, mean score	44.9 (12.6)	42.5 (12.3)	47.8 (14.9)	0.64
Emotion regulation, mean score	72.7 (18.2)	73.7 (18.8)	72.6 (19.8)	0.83
Mindfulness, mean score	39.2 (5.3)	38.9 (5.3)	39.5 (7.2)	0.40
Stress responses, mean score	67.1 (11.4)	68.2 (10.0)	66.0 (11.2)	0.68
Heart Rate, mean				
Resting	74.4 (10.7)	70.1 (11.0)	67.9 (12.6)	0.15
5 min exercise	111.7 (18.1)	104.4 (22.8)	N/A	0.49
9 min exercise	113.1 (17.2)	108.8 (22.7)	N/A	0.60
5 min meditation	68.7 (11.6)	63.1 (9.5)	N/A	0.32
9 min meditation	66.8 (10.4)	61.5 (9.8)	N/A	0.36
5 min control	N/A	N/A	69.2 (14.5)	
9 min control	N/A	N/A	68.9 (14.8)	
3 min post	75.3 (12.5)	71.5 (15.4)	67.9 (14.1)	0.46
RPE, mean	9.0 (2.4)	9.5 (1.8)	N/A	0.69
Speed, mean km/h	6.1 (2.4)	5.6 (2.7)	N/A	0.38

Values in parentheses are standard deviations; BMI = measured body mass index; Currently meditating = proportion of the group who reported having previous meditation experience who were practicing meditation at the time; Dysexecutive function = composite score from the Dysexecutive Questionnaire (DEX) [21]; Emotion regulation = composite score from the Difficulties in Emotion Regulation Scale (DERS) [22]; Last meal = time elapsed since last consuming food; Mindfulness = composite score from the Freiburg Mindfulness Inventory (FMI) [23]; MVPA = moderate-to-vigorous physical activity; N/A = not applicable; Previous meditation experience = proportion of the group who had any previous experience with meditation; Resting = resting heart rate; RPE = rating of perceived exertion using standard Borg 6–20 rating scale; Speed = selected miles per hour to walk at during treadmill walking bout; Stress responses = composite score from the Responses to Stressful Experiences Scale (RSES) [24]

**Table 2:** Baseline and post-intervention cognitive function scores.

	Group			
Cognitive Function Parameter	Walk then Meditate	Meditate then Walk	Control	*P*-Value
Identification Task				0.45
Baseline	0.7 (0.8)	0.8 (2.1)	0.5 (0.9)	
Post-Intervention	1.0 (1.3)	0.7 (0.9)	3.1 (11.7)	
Set-Shifting Task				0.29
Baseline	23.0 (14.2)	26.0 (16.1)	27.3 (15.1)	
Post-Intervention	23.3 (16.8)	24.7 (14.2)	22.2 (16.1)	
Stroop Task (ms)				
Congruent Trials				0.05
Baseline	884.6 (262.8)	841.4 (181.6)	839.7 (199.9)	
Post-Intervention	745.5 (214.7)	719.6 (126.6)	791.0 (109.9)	
Incongruent Trials				0.23
Baseline	1096.9 (409.1)	1039.4 (218.1)	1044.1 (203.4)	
Post-Intervention	928.3 (235.5)	917.9 (195.0)	971.2 (169.9)	
Control Trials				0.65
Baseline	891.8 (344.9)	862.1 (256.6)	829.3 (164.4)	
Post-Intervention	776.6 (231.1)	730.0 (135.5)	749.2 (100.0)	
Subjective Cognition				
Attention				0.75
Baseline	78.0 (20.0)	76.8 (23.1)	79.6 (18.5)	
Post-Intervention	78.1 (18.0)	80.2 (23.6)	78.5 (19.6)	
Task-Switching				0.09
Baseline	85.5 (13.8)	77.5 (18.7)	79.0 (20.9)	
Post-Intervention	75.7 (21.3)	82.3 (24.1)	77.9 (19.4)	
Flexibility				0.90
Baseline	61.4 (25.5)	54.8 (25.4)	53.5 (23.2)	
Post-Intervention	57.1 (27.1)	53.7 (26.0)	49.3 (23.4)	
Inhibition				0.57
Baseline	80.6 (22.0)	79.3 (23.1)	84.9 (18.6)	
Post-Intervention	71.7 (28.7)	61.2 (32.0)	72.8 (27.4)	
Trail Making (s)				
Trail Making A				0.50
Baseline	16.2 (4.1)	17.0 (3.8)	16.4 (4.0)	
Post-Intervention	13.8 (3.0)	13.8 (3.2)	14.1 (2.9)	
Trail Making B				0.53
Baseline	51.0 (37.3)	38.2 (7.9)	38.5 (25.0)	
Post-Intervention	39.4 (16.5)	34.1 (13.1)	32.3 (11.2)	
